# How large language models judge and influence human cooperation

**DOI:** 10.1093/pnasnexus/pgag297

**Published:** 2026-09-29

**Authors:** Alexandre S Pires, Laurens Samson, Sennay Ghebreab, Fernando P Santos

**Affiliations:** Informatics Institute, University of Amsterdam, Science Park 900, Amsterdam, XH 1098, The Netherlands; Informatics Institute, University of Amsterdam, Science Park 900, Amsterdam, XH 1098, The Netherlands; City of Amsterdam, Amsterdam, The Netherlands; Informatics Institute, University of Amsterdam, Science Park 900, Amsterdam, XH 1098, The Netherlands; Informatics Institute, University of Amsterdam, Science Park 900, Amsterdam, XH 1098, The Netherlands

**Keywords:** large language models, cooperation, indirect reciprocity, social norms, complex systems

## Abstract

Humans increasingly rely on large language models (LLMs) to support decisions in social settings. Previous work suggests that such tools can shape people’s moral judgments. However, how LLMs evaluate decisions in social dilemmas, and the long-term implications of LLM-based assessments on human cooperation—driven by indirect reciprocity, reputations, and the capacity to judge interactions of others—remain unclear. We evaluate the social norms of 21 state-of-the-art LLMs when judging more than 40,000 fictitious decisions in social dilemmas. Through an evolutionary game-theoretical model, we study prosociality in populations adhering to these norms, theoretically studying their potential impact on long-term human cooperation. We observe a remarkable agreement in LLMs when evaluating actions against opponents perceived as *good*. Yet, there is large within- and between-model variance when assessing actions toward ill-reputed individuals. Our theoretical analysis reveals that these differences can significantly impact the prevalence of cooperation within our model. Finally, we show that prompt-based interventions can steer LLM norms, particularly when these define objective goals. Our theoretical model links LLM-based advice to long-term social dynamics, highlighting the importance of designing LLM norms considering their potential effects on human cooperation.

Significance statement Large language models (LLMs) are increasingly embedded in social decision-making and have been shown to influence human behavior. Yet, their impact on human cooperation mechanisms—particularly reputation assignment guided by social norms—remains unexplored. Understanding these effects requires formalizing the social norms that LLMs employ. We assess the norms of 21 state-of-the-art LLMs when judging decisions in social dilemmas of cooperation. We find significant differences in the norms revealed between and within families of models. We find that subtle differences in LLM norms can substantially affect long-term cooperation dynamics in our model. Prompt-based interventions can partially steer LLM norms, although their effectiveness is limited and model-dependent. Our findings highlight the importance of designing LLMs with concrete social norms, considering their deployment context.

## Introduction

Large language models (LLMs) have proliferated throughout society at a remarkable speed. These tools can offer significant benefits, improving productivity, access to information, routine tasks, and education (1, 2). At the same time, LLMs suggest renewed ethical and social challenges (3, 4). Humans behave differently when interacting with LLMs than when interacting with other people (5–8). LLMs themselves can reveal biases (9, 10), based on cultural differences (11), gender (12, 13), or identity (14). Such biases also manifest when LLMs judge behaviors, potentially shaping societal norms and reinforcing existing inequalities and cultural stereotypes (15). Evidence suggests that these systems may influence our political opinions (16, 17), moral judgments (18), and expressed opinions (19). In fact, analyses of chat messages on ChatGPT show that about 1.9% of messages specifically relate to seeking personal advice and making interpersonal decisions (20). As a result, it is nowadays fundamental to understand how interactions with language models can possibly affect our very own *social fabric*, particularly our capacity to cooperate with each other on a large scale (21).

Human cooperation is a fundamental aspect of well-functioning societies, and our ability to cooperate is known to depend on shared values, interaction observability and reputation spreading (22–24). We assign reputations according to social norms, and these play a central role in deciding with whom to cooperate (25). This mechanism is known as indirect reciprocity (**IR**) (26–28). While norms and indirect reciprocity have been extensively studied in human societies (29), their role in human-AI hybrid settings remains less understood (30). Most importantly, as LLMs can influence beliefs and moral judgments, their impact in human reciprocal cooperation—and eventually human-AI prosociality (31, 32)—remains unclear. Beyond influencing norms, humans may also delegate LLMs to judge others (33, 34). In these settings, the social norms that LLMs use to assess others’ actions are highly relevant to sustain cooperation, but little is known about them.

In this work, we aim to answer three questions: (i) What social norms do LLMs adopt, within an indirect reciprocity framework? (ii) Can the social norms used by LLMs sustain cooperation under indirect reciprocity? and (iii) Can we guide the social norms of LLMs using prompt-based interventions?

To address these questions, we introduce a framework and dataset of prompts to extract the social norms used by LLMs in indirect reciprocity settings. Our dataset includes 43,200 examples of interactions, generated by combining human hand-written templates and elements. This extensive prompt dataset results from combining 16 individuals (with given names portraying different gender and cultural backgrounds), playing a 2-action game on two roles, with two possible reputations, and interacting in nine different contexts (e.g. offering money or food); all of these interactions are prompted based on five template variations (see Methods for a detailed description of the prompt dataset creation and size). In these examples, cooperation is explicitly or implicitly framed as incurring a cost (*c*) to the helper and a benefit (*b*) to the individual being helped, with b>c. We provide these examples to a total of 21 LLMs (e.g. GPT-4o (35), Deepseek R1 (36), and Grok 2 (37)) and ask them to assign a reputation to individuals that cooperate or refuse to cooperate. From the answers provided, we extract a social norm following the formalism typically considered in indirect reciprocity models.

In a second stage, we employ a traditional evolutionary game-theoretical model to evaluate the capacity of LLM-based norms to sustain human cooperation. Crucially, our model provides the flexibility to consider probabilistic norms, thereby allowing us to evaluate the average responses of LLMs and their eventual uncertainty. We study two regimes of reputation spreading: public reputations, where following an interaction, observers gossip about the assigned reputation until consensus is reached (38); and private reputations, where each agent possesses an individual view of every other agent, maximizing possible disagreement (39). Notably, cooperation under a social norm is highly dependent on gossip levels and is particularly difficult in private assessment settings (29). While private reputation systems emulate settings where individuals hold private views and might disagree on how to assess others, public reputation systems might in reality result from a large number of gossip rounds, population-wide broadcasting, and/or centralized reputation systems.

Finally, we study the impact of prompt engineering on the social norms expressed by LLMs by including additional instructions in our prompt dataset. This method allows us to measure how LLMs interpret and adopt user-defined preferences and goals. A schematic view of our pipeline is presented in Fig. 1.

**Figure 1:**
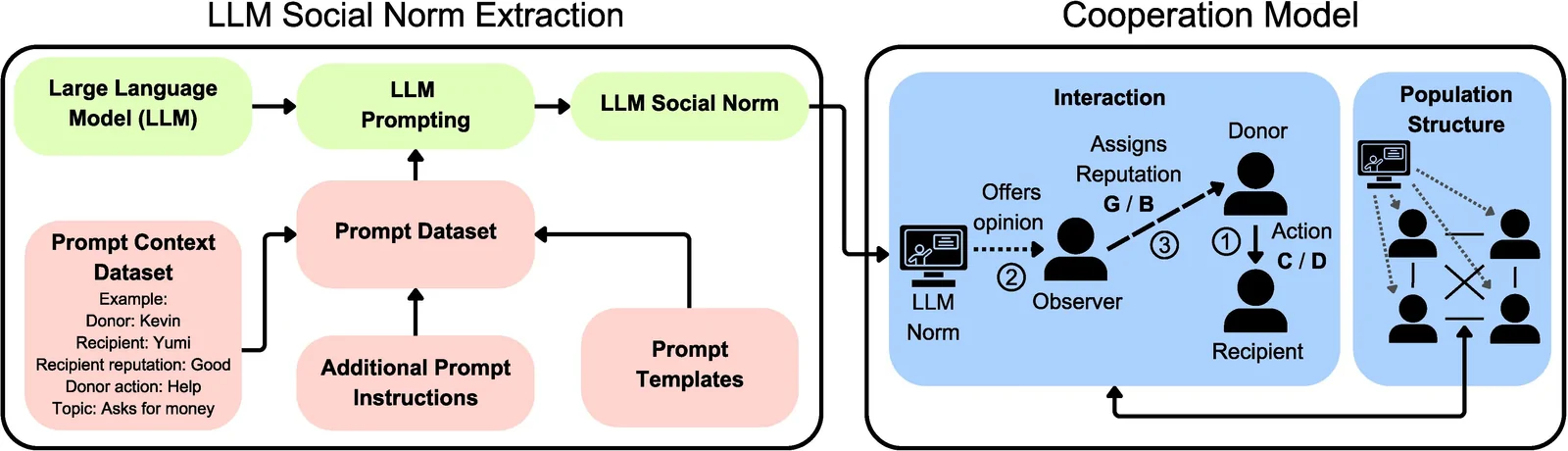
An overview of our framework to extract and evaluate LLM-based cooperation norms. We use a dataset of prompts portraying various interaction contexts to extract the social norm used by LLMs in a format compatible with models of indirect reciprocity. Norms are then evaluated through evolutionary game theory, measuring their capacity to sustain cooperation. In our model, a population of adaptive agents repeatedly plays donation games. When deciding to cooperate (C) or defect (D) with others, agents can access reputations (good, G, or bad, B). Observers then apply the LLM-inferred social norm to assign reputations to donors. This setup allows us to test the impact of LLM-based norms in long-term cooperation.

## Results

### Measuring social norms used by LLMs

We measure the social norms of multiple LLMs, following the formalism typically used in indirect reciprocity (40–42, 46, 47). We consider 21 LLMs with varying levels of accessibility (open- and closed-weights models), parameter sizes, and different price ranges, reflecting both models used by everyday consumers and enterprises (see Methods). To assess these social norms, we generate a dataset of 43,200 prompts that ask the model to assign a reputation (good or bad) to an individual (donor), after observing this donor either help or not help another individual (receiver). Importantly, the LLM is also informed about the prior reputation it assigned to the receiver, allowing (but not forcing) it to utilize reputational information, reflecting a second-order social norm (41).

In Fig. 2, we present the average assigned reputation by each LLM when asked to assess an interaction. With the exception of Llama 2 7B, all tested models assess interactions with good individuals following the same principles of norms identified theoretically as the most cooperative (known as the leading eight social norms) (48): cooperating with good individuals results in a good reputation to the donor, and defecting against good individuals is considered bad.

**Figure 2:**
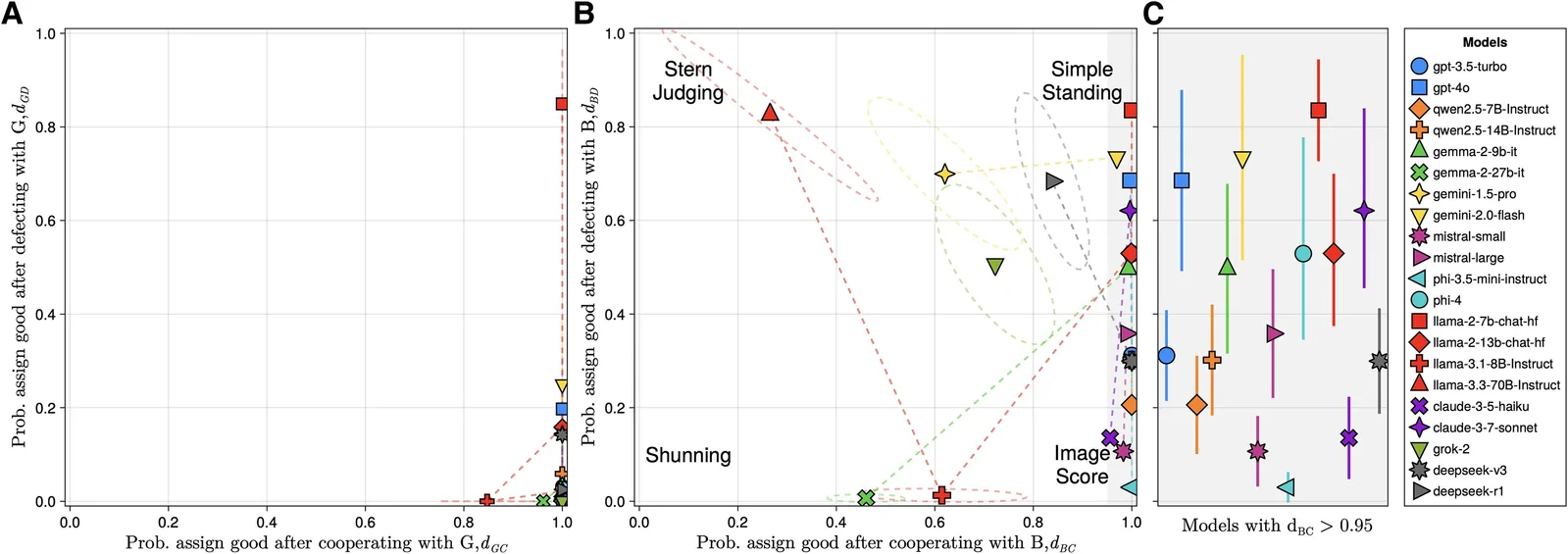
Social norms extracted from different LLMs. Each point corresponds to the average social norm from an LLM, located in a space where axes represent the probability of assigning a good reputation to the donor after cooperation (*x*-axis) or defection (*y*-axis). Ellipses indicate the covariance-defined 1 SD of the calculated norm, indicating uncertainty. Models of the same family have identical colors and are connected in order of version and parameter size. A) The result of judging interactions against opponents with good reputations. The clustered points in the bottom-right area of the plot reveal that most models agree that, when facing good recipients, cooperating is good and defecting is bad. B) When judging interactions with bad recipients, models disagree: Several models assign a good reputation to cooperators, but differ in their behavior toward defectors. Earlier models tend to consider primarily the action done by the donor, while recent model versions consider good any action against a bad individual, and therefore also consider the receiver’s reputation. This panel also represents the location of norms traditionally studied in indirect reciprocity (27, 40, 41): *Image-score* (42), *Shunning* (43), *Simple Standing* (44), and *Stern-Judging* (45). C) Uncertainty region of models that frequently assign a good reputation when observing cooperation with bad individuals ($d_{\mathrm{BC}}>0.95$, shaded gray).

When assessing interactions with bad individuals, we observe a large variation in judgments, also echoing previous theoretical works indicating that ranking the most cooperative social norms depends on subtle details such as reputation observability and behavioral errors (39, 48). Most LLMs tested assign a good reputation to those who cooperate with bad recipients, but vary in how they judge defections. This corresponds to a mixture of two social norms known to sustain cooperation: **Image Score** (**IS**, cooperating is good, defection is bad (42)) and **Simple Standing** (**SS**, cooperating is always good, defecting against bad individuals is also good (26, 44)). Notably, **IS** is a low-complexity norm, as it considers solely the donor’s action and not the reputation assigned to the recipient (41). There are also a variety of models that present different norms, such as Gemma 2 27B IT (49) and Llama 3.1 8B (50), which use a mixture of **IS** and a different social norm known as **Shunning** (**SH**), where only cooperating with good people is considered good. **SH** is, importantly, a strict norm as it labels any individual interacting with a bad recipient as bad, which can lead to a spread of bad reputations (43, 51, 52). Llama 3.3 70B (53) employs a norm close to **Stern-Judging** (**SJ**), where cooperating with good individuals is good, but agents should defect when facing bad individuals, motivating punishments (45). Finally, Gemini 1.5-Pro (54) and Grok 2 (37) both feature norms with no consistent rule for being considered good or bad when facing bad individuals.

With some exceptions, most LLM families we tested tend to move from **IS** toward **SS** as versions and parameter size increase. This indicates a shift toward higher complexity social norms (41) which make use of more context about donations’ recipients to assign a reputation. Moreover, different versions of the same family can have vastly distinct social norms, such as Claude 3.5 Haiku (55) and Claude 3.7 Sonnet (56), despite their similar ethical goals (57). The Llama family (58) presents an interesting example of version differences, as the norms of each model differ significantly. First, Llama 2 7B assigns a good reputation to donors almost independently of the recipient’s reputation, making it the simplest social norm. Its larger version, Llama 2 13B, instead uses a **SS–IS** mixture, while, as previously stated, Llama 3.1 8B and Llama 3.3 70B present entirely different norms. The only other family of models that evolves away from **SS** is Gemma, where the Gemma 2 9B model uses a **SS–IS** mixture, yet the larger 27-billion-parameter model adopts a **SH–IS** mixture, a similar trajectory to Llama 2 13B and Llama 3.1 8B.

We note that the interactions portrayed in the prompts, which LLMs are asked to evaluate, differ in phrasing, topics contextualizing the interactions, and actor names suggesting different gender and cultural backgrounds. This variability allows us to measure within-model variance and sensitivity to small variations in prompts. In Fig. 2, this uncertainty is represented by an ellipse indicating 1 SD of the calculated social norm (see Methods section). While some models, such as Claude 3.5 Haiku, showcase very low variation, indicating that their social norm is consistent across phrasings, actor names and interaction contexts, models such as Grok 2 display more volatile norms. We present an expanded analysis of these biases, and sensitivity to phrasing, in the Supplementary material. In general, we find that LLM-based assessments also depend on gender and perceived cultural background of the recipients (as inferred from their names), and more significantly, on the context of the interaction.

### Cooperation under LLM norms

After extracting the social norm revealed by different LLMs, we pose the question: how are such norms likely to impact long-term cooperation dynamics? To this end, we applied a classic evolutionary game-theoretical model to study cooperation in an adaptive population where individuals repeatedly play *donation games* and assess each other following the social norms observed in LLMs. In the donation game, one agent (donor) decides to cooperate with another agent (receiver). To cooperate means incurring a cost *c* to provide a benefit *b* to the receiver (b>c); to defect means paying no cost and providing no benefit. This simple game captures the quintessential social dilemma of cooperation: to cooperate is socially desirable, as its benefits are higher than the costs (as b>c); however selfish individuals are likely to avoid cooperating as it is individually costly (as c>0). Donors decide to cooperate or defect depending on behavioral strategies: (i) always cooperate, (ii) always defect, or (iii) cooperate only with good individuals. Crucially, strategies with a greater payoff are more likely to be imitated and used by other players (59). We assume that every nonparticipating agent will observe donation interactions and assign a reputation to the donor. Importantly, when assessing LLM norms, our model assumes a limit where all humans have access to the same LLM, and assign to every donor the reputation suggested by that LLM’s norm (see Supplementary material for results on limited LLM influence in human social norms). Following traditional models, cooperative actions have a probability to be incorrectly executed, resulting in a defection (with a probability $e_{e}$), and social norms might be incorrectly applied (with a probability $e_{a}$). Refer to the Supplementary material for an analysis of the effect of the different cooperation model parameters.

In Fig. 3, we present the prevalence of cooperation (cooperation index, *I*, see Methods) across the same norm space introduced in Fig. 2, for a public reputation setting. Following traditional results in **IR** (60), we observe that the space around **SJ** presents the highest level of cooperation (I≃0.96), with cooperation decaying approaching **SS** (I≃0.75), more so around **IS** (I≃0.51), and becoming substantially lower closer to **SH** (I≃0.24). By overlaying the social norms extracted from LLMs on this cooperation map, we observe that most models adopt the **SS-IS** edge leading to I∈[0.5,0.75]. We also find that most of the benchmarked LLM families are evolving toward **SS** (Fig. 3, top-right corner), and the capacity to promote cooperation through the social norm is improving in relation to earlier models. Interestingly, of all tested models, only Llama 3.3 70B IT exhibits a norm capable of maximizing cooperation under this scenario. Furthermore, the models with unclear social norms, such as Grok 2 and Gemini 1.5 Pro, are still capable of promoting high levels of cooperation.

**Figure 3:**
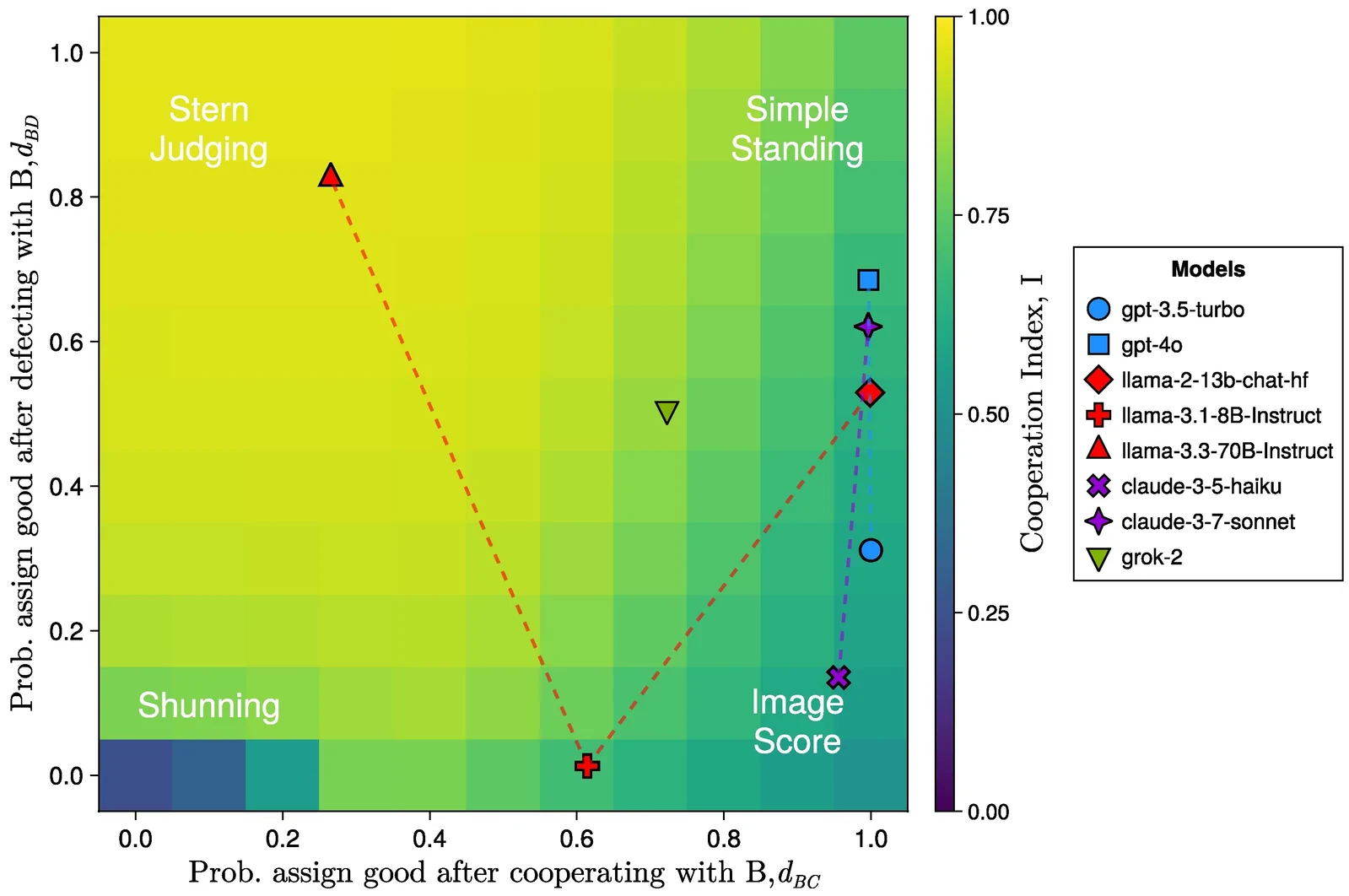
Cooperation index, *I*, across the same space of social norms as Fig. 2, measuring the average cooperation observed in a population using that social norm. Each axis corresponds to the probability of assigning a good reputation to an individual following a cooperation (x-axis) or defection (y-axis) with a bad recipient. The remainder of the norm, used when facing good recipients, is set to $d_{\mathrm{GC}}=1$ and $d_{\mathrm{GD}}=0$ (only cooperating with good individuals is good). A subset of the tested models are overlaid, showcasing their differences in cooperation. We observe that the area surrounding **Stern-Judging** (**SJ**) achieves the highest level of cooperation, followed by **Simple Standing** (**SS**). As most tested LLM families are evolving from **Image Score** (**IS**) toward **SS**, cooperation under public reputations is improving. Yet, only the norm used by Llama 3.3 70B IT can maximize cooperation. For details on computing the cooperation index, see Methods. Parameters used: $Z=100,b/c=5.0,e_{e}=e_{a}=0.01,\gamma =0.01,\beta =1$.

Figure 3 provides an overview of the norms extracted and their connection with cooperation in a setting where reputations are assumed to perfectly spread in the population or be stored in a centralized reputation system. The introduction of LLMs is, however, also likely to impact how information spreads given within- and between-model variation and the existence of decentralized systems (e.g. fine-tuned and applied locally). This variation might lead to disagreements on reputations about the same individual, a phenomenon that is well known to affect cooperation under indirect reciprocity (39, 61). In Fig. 4, we study cooperation under a selection of LLM norms while varying (i) the benefit-cost ratios of cooperation (b/c) and (ii) the extent to which reputations perfectly spread in the population and are agreed by all; under public reputations, individuals are all assumed to share the same opinion about the same individual; under private reputations, individuals hold personal views about each other and might disagree about their opinion of others. We also show how different LLMs lead to different uncertainty regions regarding cooperation. Under public reputations, we again observe that Llama 3.3 70B IT is capable of maximizing cooperation. Despite GPT-4o being apparently close to **Simple Standing**, it presents a low level of cooperation as it sometimes assigns a good reputation to donors who defect against good individuals. Notably, Grok 2, which does not follow any of the well-defined social norms, still achieves high levels of cooperation. Models such as Claude 3.5 Haiku present fairly invariable norms that result in consistent effects in cooperation, while models like GPT-4o and Grok 2 present larger uncertainty regions, highlighting the importance of consistent norms.

**Figure 4:**
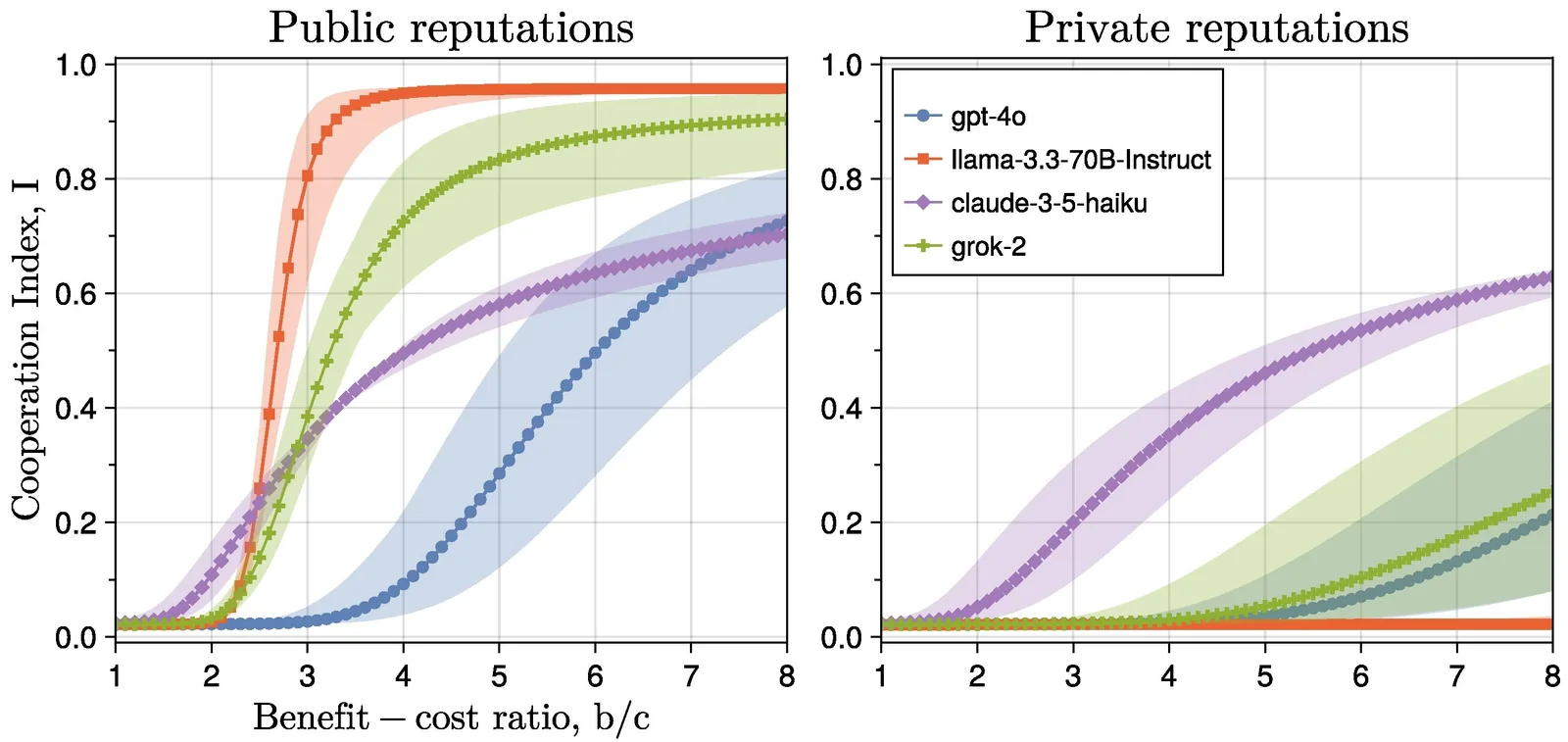
Cooperation index, *I*, using the social norm of selected LLMs, under public (left) and private (right) reputations, varying the benefit-to-cost ratio, b/c. Shaded areas represent the level of cooperation under a SD of the norm of a given LLM. We observe how cooperation under a norm is highly dependent on the centralization of reputations. Llama 3.3 70B achieves the highest cooperation among the shown norms in public reputations, but near-zero cooperation in private reputations, as it aligns with **Stern-Judging**. On the other hand, as Claude 3.5 Haiku is close to **Image Score**, although only moderately cooperative, it is more robust to private reputations. Parameters used: $Z=100,e_{e}=e_{a}=0.01,\gamma =0.01,\beta =1$.

We also observe how cooperation depends on reputation observability, with the best performing norm under public reputations (revealed by Llama 3.3 70B) presenting the worst level of cooperation under private reputations. In contrast, norms close to **Image Score**, common in earlier and smaller models such as Claude 3.5 Haiku, instead show a moderate level of cooperation, but a high resilience under decentralized reputation systems. Finally, a summary of the effects of each model parameter on cooperation guided by an LLM norm is presented in Table 1, showing how parameters that increase noise, such as higher errors and mutation rates, generally lower cooperation across all LLM social norms.

**Table 1.** Summary of the effect of varying key model parameters in cooperation rates, for social norms extracted from different LLMs.

	GPT-4o	Llama 3.3 70B IT	Claude 3.5 Haiku	Grok 2
Population size, *Z*	↓	↑	↑	↑
Execution error, $e_{e}$	↓	↓	↓	↓
Assessment error, $e_{a}$	↓	↓	↓	↓
Mutation rate, *γ*	↓	↓	↓	↓
Selection strength, *β*	↓	↑	↓	↓

An up arrow indicates that, in general, cooperation increases if the parameter increases (positive correlation). Conversely, a down arrow conveys a negative correlation. Summary based on the results detailed in Supplementary material (Fig. S8).

### Aligning social norms used by LLMs

After associating cooperation with social norms extracted from LLMs, a fundamental question follows: can we align the extracted norms to achieve cooperative states? This allows us to not only assess the flexibility of an LLM to other types of judgment rules, but the capacity of the LLM to translate user goals and expectations to changes in its social norm. In particular, we focus on four types of instructions: **universalization** (62), that asks the LLM to consider what would happen to cooperation if everyone followed its judgment rule; **empathizing** (63), which prompts the LLM to consider what it would have done if it were the donor; **signaling** (64), which suggests to consider if the assigned reputation rewards cooperation while discouraging defection; and **motivation** (65), where we instruct the LLM to consider that the reputation it assigns could affect the choices of others and that its goal is to maximize cooperation.

In Fig. 5, we present how the norms extracted from a subset of the LLMs change when using these additional instructions. Starting with **empathizing**, the change in norm is highly model dependent. While Llama 3.1 8B IT and, to a lower degree, Gemini 1.5 Pro shift in the direction of **Shunning (SH)**, suggesting a low level of empathy toward the donor, Phi-4 instead shifts to **Simple Standing (SS)**, indicating high empathy. On the other hand, Qwen 2.5 7B IT more closely follows **Image Score (IS)**. When prompted to maximize cooperation via **motivation**, the tested models universally shift their social norm toward **IS**, indicating an association that punishing defectors but not cooperators, regardless of the receiver’s reputation, is more likely to lead to high cooperation. Under **signaling**, all models reduce the probability of assigning a good reputation to defectors (in Llama 3.1 8B IT this probability was already close to 0), thereby effectively signaling that defections are negative. Finally, **universalization** is again model-dependent. While Gemini 1.5 Pro and Llama 3.1 8B IT move toward **SH** (with the latter assigning “bad” in every scenario, including with good recipients), Phi-4 and Qwen 2.5 7B IT both approach **IS**.

**Figure 5:**
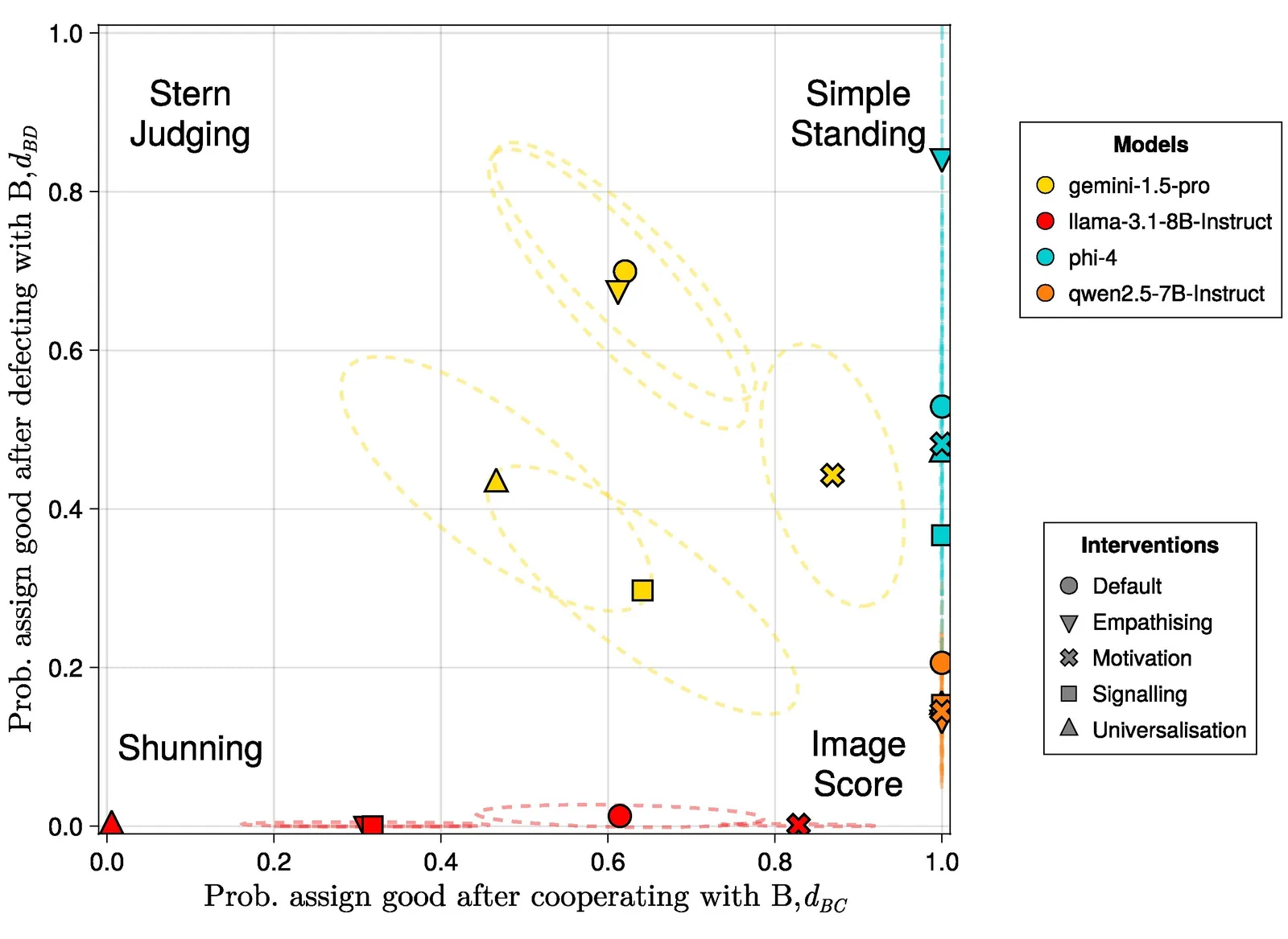
The impact of prompt-based interventions in steering LLM-based cooperation norms. The obtained norms are represented in a similar space as Fig. 2B. Each point corresponds to the extracted social norm of an LLM, without (**default**) and with additional instructions to guide their reasoning. We see that goal-oriented interventions lead to behaviors that generalize across models: **Motivation** consistently brings models toward **Image Score (IS)**, while **signaling** reduces the assigned reputation of defectors. In contrast, nonobjective prompts lead to model-dependent outcomes: **empathizing** shifts Phi-4 toward **Simple Standing (SS)**, yet leads Llama 3.1 8B IT to **Shunning (SH)**, and **universalization** influences Gemini 1.5 Pro toward **SH**, yet Qwen 2.5 7B IT approaches **IS**.

In addition to the impact on the norm, the sensitivity of each model to additional instructions is also of interest. Gemini 1.5 Pro and Llama 3.1 8B IT are highly influenced by the prompt interventions, while Phi-4 and, in particular, Qwen 2.5 7B IT show a reduced effect. Importantly, however, we note that all the models tested stay within the same norm region, showcasing some level of consistency in their norms.

We conclude that, depending on the model, the interventions tested are effective in shifting the social norm used by an LLM. Notably, **motivation** and **signaling** are consistent across most models, and are particularly impactful in incentivizing **IS**, which disregards reputational information about the receiver and is most successful at promoting cooperation in private reputation environments (see Fig. 4).

## Discussion

Large language models (LLMs) are increasingly accessible (3) and widely used as a source of information and advice (66, 67). These models are capable of influencing the opinions of users (16) regarding how to judge others, by providing advice or deciding on their behalf (34). This raises questions regarding LLMs’ capacity to alter human reputation dynamics and affect human prosociality in the long-run.

We developed a framework and prompt dataset to assess the social norms used by LLMs in a format compatible with previous work on indirect reciprocity (**IR**), and studied how LLM social norms may affect human cooperation under an evolutionary game-theoretical model. We analyze multiple state-of-the-art LLMs, together with less powerful and more generally accessible models. We observe that, just as norms vary across human societies (68), LLMs reveal a wide range of assessments when judging human cooperation. Importantly, we observe that across versions of the same LLM family, earlier and smaller models tend to adopt a low-complexity and objective norm similar to **Image Score** (**IS**, cooperation is good, defection is bad) (69). In contrast, recent versions, with more parameters, tend to adopt more complex and reputation-dependent norms—often **Simple Standing** (**SS**, cooperation is always good, it is also good to defect against bad individuals) (44). Importantly, the social norms used across models of the same family can change drastically, such as in the Llama (58) or Claude (55, 56) family.

We further show that LLM-inferred norms can be suboptimal at maintaining cooperation, contingent on the level of information sharing in the population, under a classic evolutionary game-theoretical model where a population of individuals repeatedly plays a donation game and attribute reputations using a given LLM’s social norm. Under public reputations, a common assumption where reputations are centralized, more complex norms closer to **Stern-Judging** (**SJ**, cooperating with good individuals is good, but one should defect against bad individuals) (45) maximizes cooperation in our model by promoting the stability of individuals channeling cooperation to those also cooperating. Of the LLMs tested, we found that only Llama 3.3 70B IT utilized such a norm. Nevertheless, most evaluated families of LLMs are moving toward **Simple Standing**, and achieve higher cooperation relative to earlier LLMs, in our model. On the other hand, when reputations are private and there is no mechanism for widespread agreement, only norms close to **Image Score** maintain moderate levels of cooperation within our model. Although this scenario is less common, norms used by earlier LLMs lead to better cooperation outcomes in our framework, as they disregard prior receivers’ reputations and thus minimize disagreements.

Finally, we examined whether the social norms expressed by LLMs can be guided through prompt interventions. We tested four types of instructions, based on previous mechanisms that have been shown to improve cooperation in either LLMs or humans: encouraging the model to consider what would happen if all agents judged similarly to it (**universalization**) (62); prompting it to adopt the perspective of the donor (**empathizing**) (63); instructing it to assign a reputation that clearly promotes cooperation and discourages defection (**signaling**) (64); and providing the goal of maximizing cooperation (**motivation**) (65). Among these, **signaling** and **motivation** produced the most consistent effects across models. **Motivation** shifted LLM norms toward **Image Score**, while **signaling** lead to models more often judging defection with bad individuals as bad. This consistency may be due to the explicit goal present in the prompt, helping models organize their reasoning more coherently, akin to chain-of-thought prompting (70, 71). In contrast, the **empathizing** and **universalization** interventions yielded model-dependent outcomes. Our results extend the discussion of reasoning, observation and information-spreading cooperation mechanisms, such as signaling (64) and empathy (8, 63, 72), by showing how LLMs interpret such mechanisms and consequently alter their social norms.

Although we focus our analysis on cooperation dynamics, we also identify biases in how LLMs assign reputations. Models differ in their social norms depending on the gender and region of the actors in the prompt (as inferred from their names), aligning with previous results on these types of biases (13, 14, 73). We find a high variation in the norms displayed depending on the context of the interactions observed by the LLMs, with a greater uncertainty in ambiguous prompts (e.g. “Liam needs help.”) than in more defined contexts (e.g. “Alice needs money to eat.”), again aligning with previous works (74). While this variation may reflect contextual sensitivity, it also introduces unpredictability in reputation judgments, which can potentially lead to unintended social biases when LLMs are used in real-world decision-making processes. In our model, we quantify these variations in social norms as uncertainty regions in the space of possible social norms. We show how some models, such as Claude 3.5 Haiku (55) and Mistral Small (75) exhibit low uncertainty, and therefore a prompt-resilient and consistent social norm. On the other hand, models such as Grok 2 (37) and Llama 3.3 70B (53) use norms that are highly sensitive to actor names, phrasing and contexts for interactions.

Our results highlight an important concern: although LLMs are trained on vast amounts of human-generated data, the norms revealed by these tools vary significantly from each other and, according to our model, may fail to maintain human cooperation. Given the increasing integration of LLMs in decision-making processes, ranging from content moderation to AI-assisted governance (76), it is crucial to consider not only their implicit social norms, but also their capacity to influence human cooperative behavior (33), including through indirect means such as altering human reputation assignments or delegation. Previous work has shown that LLMs can persuade humans (77, 78), shaping their beliefs and behaviors. The risk that these effects may be augmented in a future where *agentic AI*, powered by LLMs, becomes highly customizable and acts autonomously on behalf of humans motivates the need to understand LLM-influenced human cooperation dynamics (79).

The stylized model we develop presents limitations due to our simplifying assumptions. First, we assume that humans will be influenced to use the social norm suggested by the LLMs, which remains to be empirically verified. While prior work suggests that LLMs can, in fact, influence human judgments (16), it remains under-explored whether these results extend to individuals assessing social dilemmas of cooperation. Furthermore, we considered scenarios where only one LLM is present and equally accessible to everyone. A deeper analysis could consider inequalities in LLM access and scenarios with multiple distinct LLMs. Finally, our analysis is theoretical and, for mathematical tractability, assumes a homogeneous population of individuals. These limitations suggest future work on theoretical modeling (e.g. allowing for different segments of the population to be influenced by different LLM versions) and experimental work (e.g. testing the external validity of our model and the extent to which LLM advice on judging cooperative behavior is followed by users).

Despite these challenges, our work offers a means of combining recent LLM tools and prior literature on human cooperation through reciprocity. This answers a call for better integration of (recent) LLM research within (traditional) multiagent systems literature (80). Overall, by combining LLM-extracted behaviors and evolutionary dynamics modeling, our framework provides an example of long-term behavioral dynamics analysis in societies where LLM-based advice becomes prevalent. Our results and conclusions are constrained to current LLM models and our game-theoretical model. However, we hope that our framework can be used as a benchmark to audit the development of future LLMs, acknowledging their potential influence on human cooperation when used as decision-making advice tools—a critical task to address given reported effects such as *human-AI feedback loops* (81) and *data poisoning* (82). Crucially, we already show that subtle differences in LLM model versions and prompting lead to variation in the social norms extracted, and such differences significantly affect long-term cooperation dynamics within our theoretical framework.

## Methods

We study LLM norms and their effect on human cooperation following a two-step pipeline: First, determining the social norm used by the LLM; and second, studying the impact of this social norm on human cooperation via an evolutionary game-theoretical indirect reciprocity (**IR**) model.

The model, described in the Section Cooperation model, is used to determine the cooperation level of an adaptive population playing the donation game, using a given social norm. In our model, we make use of the social norms extracted from LLMs.

To assess the social norm employed by the LLM, a prompt dataset is generated from a template system, allowing many prompt variations of the same structure. The LLM is then individually prompted on each prompt of the dataset, and its answers are parsed and aggregated into a social norm. This process is described in Section LLM social norm assessment.

### Cooperation model

Following traditional models of **IR** (39, 83), we consider a finite and well-mixed population of *Z* adaptive agents repeatedly playing a donation game. In this game, two agents are paired, with one being in the role of donor, and another as receiver. The donor can play one of two actions: **C**, cooperate, which gives a benefit *b* to the receiver at its own cost of *c*, (b>c>0); or **D**, defect, where no benefit is given, at no cost to the donor. Each individual has assigned a binary reputation, either good (**G**) or bad (**B**), to every other individual in the population. We further assume that all individuals not participating in a game observe the interaction, assigning a reputation to the donor. The details of the reputation assignment are explained in Section Reputation dynamics.

At each game, the action picked by the donor is dependent on its strategy. We consider strategies conditioned on the reputation of the receiver, which we encode as $s=(s_{G},s_{B})$, where $s_{G}$ and $s_{B}$ are the probability of picking action **C** against an individual seen as **G** and **B**, respectively. We focus our analysis on three commonly studied pure strategies: *ALLC*  (1,1), which always cooperates; *ALLD*  (0,0), which always defects; and *DISC*  (1,0), which only cooperates with **G** individuals and defects otherwise. Additionally, we consider errors in the execution of the strategy, where with a probability $e_{e}$ a donor intending to cooperate will instead defect (84, 85).

#### Reputation dynamics

The assignment of reputations is made individually by each agent, following a social norm common to the entire population. In particular, we consider second-order social norms (41), where the assigned reputation of the donor is dependent on its action and the reputation of the receiver, in the view of the observer. We formalize a social norm as $d=(d_{G,C},d_{G,D},d_{B,C},d_{B,D})$, $d\in [0,1{]}^{4}$, where each entry corresponds to the probability of assigning a good reputation given the donor’s action and the receiver’s reputation. We include assessment errors, where with a probability $e_{a}$ an agent assigns the opposite reputation to what was intended (86). In our figures we explicitly identify four key norms, previously discussed in the literature (48, 52): **Image Score (IS)** (42), d=(1,0,1,0), where cooperation is good and defection is bad; **Simple Standing (SS)**, d=(1,0,1,1), where only defection against good agents is bad, and any other action is good; **Shunning (SH)**, d=(1,0,0,0), where it is only good to cooperate with good agents; and **Stern-Judging (SJ)** (45), d=(1,0,0,1), where it is only good to cooperate with good agents and defect with bad agents. Importantly, this norm formulation accounts for continuous values, $d\in [0,1{]}^{4}$, a detail that allows us to directly test average norms extracted from LLMs.

Given a social norm *d*, the probability of an observer effectively assigning a good reputation to a donor who will use action Y∈{C,D} against a receiver with reputation X∈{G,B}, considering errors, is given by $P_{X,Y}$ (47):


(1) $$\begin{array}{rl} P_{G,C}^{d} & =d_{G,C}(\epsilon -e_{a})+d_{G,D}(1-\epsilon -e_{a})+e_{a}\\ P_{G,D}^{d} & =d_{G,D}(1-2e_{a})+e_{a}\\ P_{B,C}^{d} & =d_{B,C}(\epsilon -e_{a})+d_{B,D}(1-\epsilon -e_{a})+e_{a}\\ P_{B,D}^{d} & =d_{B,D}(1-2e_{a})+e_{a} \end{array}$$

where $\epsilon =(1-e_{e})(1-e_{a})+e_{e}e_{a}$, the probability that no errors occur, or both types of error occur simultaneously.

The reputation of each strategy is dependent on the current strategies in the population, yet the payoffs of each strategy are also linked with the reputations of each strategy. We adopt the common assumption that reputations change at a faster timescale than strategies (39, 83). This allows us to consider the convergence of reputation dynamics at any strategy state $n=(n_{ALLC},n_{ALLD},n_{DISC})$, where $n_{s}$ represents the number of agents using strategy s∈S={ALLC,ALLD,DISC}, and later analyze the dynamics between strategy states (see Section Strategy dynamics).

In a well-mixed population, at any strategy state n, the average reputation of each strategy can be represented as $r_{s},s\in S$, the probability that an agent using strategy *s* is considered good by a randomly sampled individual. These can be approximated by the following set of ordinary differential equations (86):


(2) $$\{\frac{dr_{s}}{dt}=g_{s}(t)-r_{s}(t),\;s\in S,$$

where $g_{s}(t)$ is the probability that an individual will assign a good reputation to an agent using strategy *s*, at time step *t*. For each strategy, these are given as (87):


(3) $$\begin{array}{rl} g_{ALLC}(t) & =r(t)P_{G,C}^{d}+\left(1-r(t)\right)P_{B,C}^{d}\\ g_{ALLD}(t) & =r(t)P_{G,D}^{d}+\left(1-r(t)\right)P_{B,D}^{d}\\ g_{DISC}(t) & ={\tilde{q}}^{g}P_{G,C}^{d}+{\tilde{q}}^{d}\left(P_{G,D}^{d}+P_{B,C}^{d}\right)+{\tilde{q}}^{b}P_{B,D}^{d} \end{array}$$

where $r(t)=\sum_{s\in S}(n_{s}/Z)\cdot r_{s}(t)$ is the average reputation in the population. Furthermore, ${\tilde{q}}^{g}$, ${\tilde{q}}^{b}$, are the probability of two individuals agreeing that a focal individual is considered good and bad, respectively, and ${\tilde{q}}^{d}$ the probability that the two agents disagree on the assigned reputation. Before any gossip has occurred, and as such reputations are private, these are given by:


(4) $$\begin{array}{cc} q^{g}=\sum_{s\in S}\frac{n_{s}}{Z}r_{s}^{2} & q^{b}=\sum_{s\in S}\frac{n_{s}}{Z}(1-r_{s}{)}^{2}\\ q^{d}=\sum_{s\in S}\frac{n_{s}}{Z}r_{s}(1-r_{s}) \end{array}.$$

In order to study the influence of LLMs under different reputation sharing settings, we vary the time allowed for gossip to spread. We consider *T* rounds of gossip, where at each round a randomly picked individual adopts the reputations assigned by another individual (87). Considering the size of the population, this is normalized as the gossip duration, t=T/Z. At t=0, reputations are private and disagreements are maximized, as no gossip occurs, and at t=∞, reputations are public, and no disagreements are present. After gossip, the probability of agreements and disagreements in the assigned reputations of a focal individual are given by


(5) $$\begin{array}{rl} {\tilde{q}}^{g} & =q^{g}+q^{d}\cdot (1-e^{-t})\\ {\tilde{q}}^{b} & =q^{b}+q^{d}\cdot (1-e^{-t})\\ {\tilde{q}}^{d} & =q^{d}\cdot e^{-t} \end{array}.$$

#### LLM norm adoption

In our model, LLMs always play the role of observers, and are accessible by all individuals. We consider the simplest scenario, where we assume any agent will consult an LLM and fully adopt its opinion. As such, if the LLM presents a social norm $d^{L}$, a norm is extracted via the process described in Section LLM social norm assessment, the social norm used by the population will be $d=d^{L}$.

#### Strategy dynamics

Having the average reputation at each strategy state, we next define how the population transitions between states. We employ a birth–death process using two replication mechanisms: mutations, where an agent adopts a random strategy with probability *γ*, and imitations, where an agent may adopt the strategy of another. Imitation is modeled using the *pairwise comparison rule* (59), also known as the *Fermi update rule*: the probability that an agent using strategy *s* imitates another using strategy $s^{\prime }$ is equal to $P_{s\to s^{\prime }}(n)=(1+e^{-\beta \Delta F_{s,s^{\prime }}}{)}^{-1}$, where $\Delta F_{s,s^{\prime }}(n)={\bar{F}}_{s^{\prime }}(n)-{\bar{F}}_{s}(n)$ is the difference between the average fitness of strategy $s^{\prime }$ and strategy *s*, and *β* is the strength of selection. Strategies with higher fitness difference are more likely to be imitated. A higher strength of selection (β→∞) results in a deterministic evolutionary process, while lower values (β→0) tend toward a random selection process.

In the donation game, the average fitness of a strategy *s* is given by $F_{s}(n)=bR_{s}(n)-cD_{s}(n)$, having two factors: $R_{s}(n)$ the probability that an individual using strategy *s* is cooperated with, therefore obtaining a benefit *b*, and $D_{s}(n)$, the probability that an individual using strategy *s* will donate, incurring a cost *c*. The first is given by


(6) $$R_{s}(n)=(1-e_{e})\left(\frac{n_{ALLC}}{Z}+\frac{n_{DISC}}{Z}r_{s}\right),$$

while the probability of donating is calculated as


(7) $$\begin{array}{c} D_{s}(n)=\left\{ \begin{array}{cc} 1-e_{e}, & \text{if }s=ALLC\\ 0, & \text{if }s=ALLD\\ (1-e_{e})r, & \text{if }s=DISC \end{array}\right. \end{array}.$$

Having the average fitness at each state, we define a Markov chain containing all possible strategy states to study the adoption of strategies over time (88). The total state space is given by $M=\{n=(n_{i},n_{j},n_{k})|n_{i}+n_{j}+n_{k}=Z\}$, with a total of $S=\left(\begin{array}{c} Z+2\\ 2 \end{array}\right)$ states. For any two states differing by the strategy of a single agent, using strategy *s* and $s^{\prime }$, respectively, the transition probability will be equal to the probability of a mutation or imitation occurring:


(8) $$M_{s\to s^{\prime }}(n)=(1-\gamma )\frac{n_{s}}{Z}\frac{n_{s^{\prime }}}{Z-1}P_{s\to s^{\prime }}(n)+\gamma \frac{n_{s}}{2Z}.$$

The transition matrix of the strategy Markov chain, *M*, where each entry $M_{a,b}$ corresponds to the probability of transitioning from state $n^{a}$ to state $n^{b}$, is given by


(9) $$M_{a,b}=\left\{ \begin{array}{cc} M_{s\to s^{\prime }}(n^{a}) & \text{if }n_{s}^{b}=n_{s}^{a}-1\wedge n_{s^{\prime }}^{b}=n_{s^{\prime }}^{a}+1\\ & \wedge \;n_{s^{\prime \prime }}^{b}=n_{s^{\prime \prime }}^{a}\\ 1-\sum M_{s\to s^{\prime }}(n^{a}) & \text{if }n^{b}=n^{a}\\ 0 & \text{otherwise} \end{array}\right.,$$

where $s,s^{\prime },s^{\prime \prime }\in S$ and $s\neq s^{\prime }\neq s^{\prime \prime }$. Since *M* is irreducible (assuming γ>0 and finite *β*), we can calculate its stationary distribution *σ*, as it is unique and equal to the eigenvector associated with the eigenvalue 1 (89), resulting in σM=σ. We define $\sigma_{n}$ as the value of the stationary distribution at state n.

#### Cooperation index

To quantify the frequency of cooperation in the population, we employ the cooperation index (83), corresponding to the probability of observing a donation at any interaction, given by


(10) $$I=\sum_{n\in M}\sigma_{n}\frac{1}{Z}\left(D_{ALLC}(n)\cdot n_{ALLC}+D_{DISC}(n)\cdot n_{DISC}\right),$$

which measures cooperation at each strategy state, weighted by its frequency in the stationary distribution of the strategy Markov chain.

### LLM social norm assessment

We next detail the assessment process of the social norm of an LLM. First, we outline the construction of the prompt dataset, followed by the method to process responses and aggregate them to define a social norm. The models we test include: GPT-3.5-Turbo and GPT-4o (35), Qwen 2.5 7B IT and Qwen 2.5 14B IT (90), Gemma 2 9B IT and Gemma 2 27B IT (49), Gemini 1.5 Pro (54) and Gemini 2.0 Flash (91), Mistral Small (75) and Mistral Large (92), Phi-3.5 Mini IT (93), and Phi-4 (94), Llama 2 7B and Llama 2 13B (58), Llama 3.1 8B IT (50), and Llama 3.3 70B IT (53), Claude 3.5 Haiku (55) and Claude 3.7 Sonnet (56), Grok 2 (37), Deepseek V3 (95), and Deepseek R1 (36), where “B” reflects the parameter size of the model, in billions, and IT refers to an instruction-tuned version of the model. To ensure reproducibility, all models are queried using a temperature of zero. The details of each model are available in the Supplementary material.

#### Prompt dataset

The prompt dataset is constructed to assess what social norm an LLM is using. This is done by providing LLMs the context of a donation game interaction, and requesting them to output an assessment regarding the reputation of the donor.

As detailed in Section Cooperation model, we make use of second-order social norms, where the reputation of a donor depends on its action (**C** or **D**) and on the reputation of the receiver (**G** or **B**). As such, each prompt first presents the donor and receiver to the LLM, together with the prior reputation of the receiver, followed by a description of their interaction and the action chosen by the donor. Finally, the LLM is instructed to provide its new reputation, answering exclusively “good” or “bad.”

To ensure variety in the dataset, a template system was used with 5 template prompts, presenting the structure detailed above but varying in phrasing, containing fields (e.g. the donor’s action) to be filled. Various possible elements were then defined, containing actor names that suggest different genders and cultures to be used for the donor and receiver, the available actions for the donor, as well as different contexts for interactions, such as the donor asking for money or food. Importantly, we assume that gender and cultural backgrounds can be conveyed through the first names of the actors. With two names per gender per world region (four regions), for both the donor and receiver (which cannot be equal), two possible actions for the donor, two possible reputations for the recipient, nine contexts of interaction, and five variations in prompt template, a total of $((2\times 2\times 4{)}^{2}-(2\times 2\times 4))\times 2\times 2\times 9\times 5=43,200$ unique prompts were generated, resulting in 10,800 prompts per social norm entry.

Following work on prompting techniques to promote cooperation when using LLMs (62), we also study 4 types of prompt interventions by adding specific instructions to the prompt, resulting in 4 additional datasets: “Universalization” (62)—which suggests to the LLM to consider what would happen to cooperation if everyone assigned reputations like it did; “Empathizing” (63)—which asks the LLM to consider what it would have done if it was in the same situation as the donor; “Signaling (64)—which instructs the LLM to consider if the opinion it assigns clearly rewards cooperative behaviors and discourages non-cooperative behaviors; and “Motivation” (65)—which prompts the LLM to consider that the assigned reputation can potentially affect the choices of others to help, and that its goal is to maximize cooperation. The full details of all the datasets are available in the Supplementary material.

Formally, we define $D^{\prime }$ to be the dataset composed of all the prompts detailed above, for a given dataset variation. From there, a subset of the dataset is defined as $D^{f}=f(D^{\prime })$, where *f* is a filtering function that returns a subset of the dataset that matches some given criteria (e.g. the donor’s action). Our final set of datasets, T is composed of $D^{\prime }$ as well as subsets corresponding to different pairs of donor and receiver genders, name regions, and contexts of interaction. Finally, we define $D_{X,Y}$ as the set of prompts in a dataset *D* where the donor executes action Y∈{C,D}, and the receiver has a previous reputation X∈{G,B}.

#### Norm aggregation

After prompting an LLM on the prompt dataset, we next parse each of its answers. Although our prompts present formatting instructions, not all models adhere to the required format, potentially presenting alternative answers, such as “neutral,” or appending justifications after the answer. For each response, a reputation value o∈[0,1] is assigned depending on the content of the reply, with 1 and 0 corresponding to assigning the donor a good and bad reputation, respectively.

We parse solely the first paragraph with content of each response, omitting any discussion or justification by the model. If the answer contains “good” and not “bad,” 1 is assigned. If it contains exclusively “bad,” 0 is assigned. An additional rule is used to account for formatting errors: answers with “neutral” assign 0.5. Any other answer is considered invalid, and the total invalid answer rate is recorded (presented in the Supplementary material).

For each dataset D∈T, a social norm is composed by evaluating the average value of *o*, ${\bar{o}}^{D}$, of all valid answers at each pair of actions and reputations. Using dataset D, the social norm of an LLM is given by


(11) $$d^{D}=\{{\bar{o}}^{D_{G,C}},{\bar{o}}^{D_{G,D}},{\bar{o}}^{D_{B,C}},{\bar{o}}^{D_{B,D}}\}.$$

We present our primary results for $d^{L}=d^{D^{\prime }}$. However, by defining a single social norm, we omit potential uncertainty. To this end, we model the norm uncertainty using a multivariate Gaussian distribution centered at $d^{D^{\prime }}$. The covariance matrix for this distribution is estimated as the weighted covariance of $d^{D},D\in T\setminus \{D^{\prime }\}$ weighted by |D|. To assess the impact of this uncertainty on cooperation, we evaluate the cooperation index at the points lying 1 SD away from the mean ($d^{D^{\prime }}$) along each principal axis of this uncertainty distribution, reporting the maximum and minimum cooperation indices at these points.

## Supplementary Material

pgag297_Supplementary_Data

## Data Availability

All data needed to evaluate the conclusions in the article are present in the article and/or the Supplementary material. The code used is available on https://github.com/alexandresilvapires/LLM-Norm-Extraction.

## Preprints

A preprint of this article is published at arXiv:2507.00088.
